# Optimization of a Monopolar Electrode Configuration for Hybrid Electrochemical Treatment of Real Washing Machine Wastewater

**DOI:** 10.3390/ijms26136445

**Published:** 2025-07-04

**Authors:** Lidia C. Espinoza, Angélica Llanos, Marjorie Cepeda, Alexander Carreño, Patricia Velásquez, Brayan Cruz, Galo Ramírez, Julio Romero, Ricardo Abejón, Esteban Quijada-Maldonado, María J. Aguirre, Roxana Arce

**Affiliations:** 1 Centro de Nanotecnología Aplicada, Facultad de Ciencias, Ingeniería y Tecnología, Universidad Mayor, Santiago 8580701, Chile; lidia.espinoza@umayor.cl; 2 Escuela de Ingeniería en Medio Ambiente y Sustentabilidad, Facultad de Ciencias, Ingeniería y Tecnología, Universidad Mayor, Santiago 8580701, Chile; 3 Departamento de Ciencias Químicas, Facultad de Ciencias Exactas, Universidad Andres Bello, Av. República 275, Santiago 8370146, Chile; angelica.llanos@usach.cl (A.L.); marjorie.cepeda@unab.cl (M.C.); alexander.carreno@unab.cl (A.C.); patricia.velasquez@unab.cl (P.V.); 4 Laboratory of Organometallic Synthesis, Center of Applied Nanosciences (CANS), Facultad de Ciencias Exactas, Universidad Andrés Bello, República 330, Santiago 8370186, Chile; 5 Departamento de Química de Los Materiales, Facultad de Química y Biología, University of Santiago de Chile (USACH), Av. L.B. O’Higgins 3363, Santiago 9170022, Chile; brayan.cruz@usach.cl (B.C.); maria.aguirre@usach.cl (M.J.A.); 6 Departamento de Química Inorgánica, Facultad de Química y de Farmacia, Pontificia Universidad Católica de Chile, Av. Vicuña Mackenna 4860, Santiago 7820436, Chile; gramirezj@uc.cl; 7 Millennium Institute on Green Ammonia as Energy Vector (MIGA), Av. Vicuña Mackenna 4860, Santiago 7820436, Chile; 8 Laboratory of Membrane Separation Processes (LabProSeM), Department of Chemical Engineering, University of Santiago de Chile (USACH), Av. Libertador Bernardo O’Higgins 3363, Santiago 9170019, Chile; julio.romero@usach.cl (J.R.); ricardo.abejon@usach.cl (R.A.); 9 Laboratory of Separation Processes Intensification (SPI), Department of Chemical and Bioprocess Engineering, University of Santiago de Chile (USACH), Av. Libertador Bernardo O’Higgins 3363, Santiago 9170019, Chile; esteban.quijada@usach.cl

**Keywords:** electrocoagulation, electro-oxidation, monopolar electrode configuration, domestic wastewater treatment, dark greywater, optimization, hybrid electrochemical process

## Abstract

This study focuses on the design and optimization of a monopolar electrode configuration for the hybrid electrochemical treatment of real washing machine wastewater. A combined electrocoagulation (EC) and electro-oxidation (EO) system was optimized to maximize pollutant removal efficiency while minimizing energy consumption. The monopolar setup employed mixed metal oxide (MMO) and aluminum anodes, along with a stainless steel cathode, operating under controlled conditions with sodium chloride as the supporting electrolyte. An applied current density of 15 mA cm^−2^ achieved 90% chemical oxygen demand (COD) removal, 98% surfactant degradation, complete turbidity reduction within 120 min, and pH stabilization near 8. Additionally, electrochemical disinfection achieved <2 MPN/100 mL, with no detectable phenols and the presence of organic anions such as oxalate and acetate. These results demonstrate the effectiveness of an optimized monopolar EC–EO system as a cost-efficient and sustainable strategy for wastewater treatment and potential water reuse. Further studies should focus on refining energy consumption and monitoring reaction by-products to enhance large-scale applicability.

## 1. Introduction

Domestic wastewater, generated from human activities, is classified into blackwater and greywater. Blackwater comes from toilets and urinals, while greywater originates from faucets, washing machines, showers, and sinks. These waters contain dyes, surfactants, detergents, oils, fats, metals, contaminants of emerging concern (CECs), and pathogenic microorganisms [1,2,3]. Their composition varies by country, culture, lifestyle, and climate [4,5,6]. With increasing wastewater volumes and freshwater scarcity, effective treatment technologies are essential. Since blackwater carries more infectious agents [7,8], its reuse requires greater precautions. In contrast, greywater is safer and can be reused for ornamental irrigation and toilet flushing after simple treatment.

Greywater is classified into light greywater (from bathtubs, showers, and sinks), dark greywater (from laundry and kitchen sinks), and mixed greywater (a combination of both) [9]. Electrochemical Advanced Oxidation Processes (EAOPs) are promising technologies for greywater treatment [10,11,12,13,14,15,16]. These offer advantages such as environmental compatibility, versatility, scalability, energy efficiency, and reduced treatment time compared to other technologies such as chemical or biological technologies [17,18]. Electro-oxidation (EO) is a widely used EAOP due to its simple implementation and lack of pH adjustment requirements for generating strong oxidants like hydroxyl radicals (•OH). Contaminants are oxidized either directly via electron transfer at the anode or indirectly by electrogenerated oxidants [19]. The effectiveness of these oxidants depends on the anodic material, which is classified as active or non-active. In both cases, water oxidation on the anode surface produces hydroxyl radicals (Equation (1)) (2.8V vs. NHE), enabling the non-selective degradation of various organic compounds [20,21].

(1) $$M+H_{2}O\to M\left(OH\right)+H^{+}+e^{-}$$

The anode’s chemical nature influences its interaction with •OH radicals. Active anodes strongly interact with these radicals, potentially forming higher metal oxides if higher oxidation states are available. In contrast, non-active anodes exhibit weaker interactions, allowing the radicals to remain on the anode surface (Equation (2)). The formation of metal oxides leads to chemisorbed active oxygen, which can further oxidize organic compounds and pathogenic agents (R) (Equation (3)) [22].

(2) $$M\left(OH\right)\to MO+H^{+}+e^{-}$$

(3) MO+R→RO+M

Active anodes include Pt electrodes, glassy carbon, and mixed metal oxides (MMO) [23,24,25], while non-active anodes include PbO_2_, SnO_2_, and boron-doped diamond (BDD) [26,27,28,29]. Studies indicate that BDD electrodes achieve higher degradation rates than MMO; however, their production requires sophisticated equipment and extreme conditions, including high temperatures and pressures [30,31].

Although EO degrades 100% of the contaminants in most cases, its effectiveness is reduced in the treatment of wastewater with high levels of suspended solids, so it is necessary to eliminate them from wastewater to take advantage of the benefits of EO. This drawback can be overcome by combining EO with another technology known as electrocoagulation (EC) [32,33], since with EC, the suspended solids present in the solution could be eliminated through the mechanisms of formation of metal cations on the anodes, formation of hydroxyl ions on the cathode, charge neutralization adsorption of the contaminants through the reaction with metal hydroxides, and the formation of H_2_ gas at the cathode produced during this process [34]. Another advantage of EC is its ability to efficiently remove color in short treatment times. Meanwhile, EO achieves high total organic load removal [35,36,37,38,39]. Therefore, combining the two enhances treatment effectiveness, making them suitable for wastewater management.

In this work we aim to treat dark greywater by combining the EC and EO processes. For this purpose, real washing machine water was enriched with (i) one of two dyes detected in household greywater according to the literature, such as methylene blue (MB, non-azo dye) and Orange II (OII, azo dye) [40], and (ii) SDS surfactant [41]. To this end, the EC and EO processes were studied individually to determine the optimal operating conditions for the combined process. Since EC is known to be highly effective in removing dyes, the MB was treated by this process, analyzing three variables: the effect of the applied current density, the type of anode and the supporting electrolyte. With EO, the effect of the supporting electrolyte and the applied current density on the removal of OII and SDS was investigated using MMO-type electrodes.

## 2. Results and Discussion

### 2.1. Treatment of MB Solutions by EC

For details of solutions A to G, see Section 3.2. One of the major advantages of EC is its ability to completely decolorize the solution in short treatment times [42,43]. In this sense, the decolorization of a solution containing 50 mg L^−1^ of MB in an electrolytic medium of NaCl (solution A) and Na_2_SO_4_ (solution B) was studied using Fe and Al as anodes to take advantage of the species produced with these materials. In the case of Fe anodes their dissolution forms Fe (OH_)2_ precipitates at pH above 5.5 (Equation (4)):

(4) $${Fe}^{2+}+2{OH}^{-}\to Fe{\left(OH\right)}_{2\left(s\right)}$$

and remain in equilibrium with Fe^2+^ up to pH 9.5 or with monomeric species such as Fe(OH)^+^, Fe(OH)_2_, and Fe(OH)_3_^−^ at higher pH values. In the presence of O_2_, the dissolved Fe^2+^ ions oxidize form an insoluble hydroxide (Equation (5)):

(5) $${4Fe}^{2+}+10H_{2}O+O_{2(g)}\to 4Fe{\left(OH\right)}_{3\left(s\right)}+8H^{+}$$

Instead, when aluminum is used as sacrificial anode in the anode, Al^3+^ is produced (Equation (6)):

(6) $${Al}_{\left(s\right)}\to {Al}_{\left(aq\right)}^{3+}+3e^{-}$$

Al^3+^ is transformed into soluble monomeric species like Al (OH)_2_^+^, Al_2_ (OH)_2_^+^ and Al (OH)_4_^−^, which are pH dependent [4] and contribute to the water treatment.

**Figure 1:**
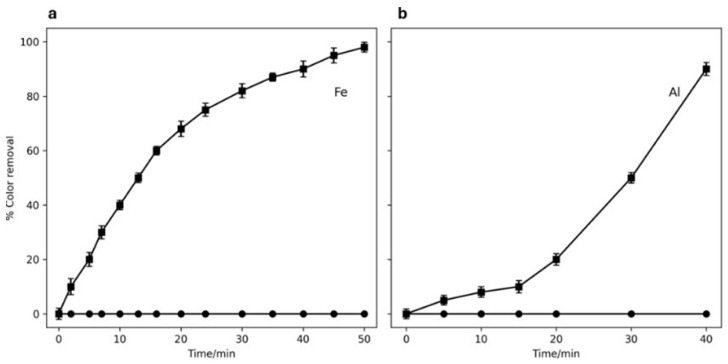
% Color removal of methylene blue using (**a**) Fe and (**b**) Al anodes. Experimental conditions = 15 mA cm^−2^, initial pH = 7.0. ● = Na_2_SO_4_ 25 mM (solution A) and ■ = NaCl 50 mM (solution B) as supporting electrolyte.

Aliquots were taken at different times of electrolysis, and according to Figure 1, complete discoloration was observed in the chloride medium, but no discoloration was observed in the sulfate medium. With the first electrolyte, electrode passivation is minimized, and oxidation is mediated by the production of active chlorine species. Likewise, color removal in this electrolytic medium is faster when Fe is used as the anode. At 30 min, 80% discoloration is obtained, attributed to the higher oxidation potential of Fe compared to Al when used as anode. The presence of chlorides in the medium inhibits or slows down the passivation of aluminum anodes, which is a typical phenomenon of this material in other electrolytic media due to the formation of a compact oxide insulating layer [44]. Due to this passivation, the generation of iron ions is nearly three times greater than that of aluminum and therefore more coagulants are expected to be formed when applying the same electrical charge. This would explain the greatest removal of the dye at short treatment times when using a Fe anode. Similar results were found in other works on MB removal using EC [45]. However, 100% removal was achieved earlier with the Al electrode (see Figure 1).

**Figure 2:**
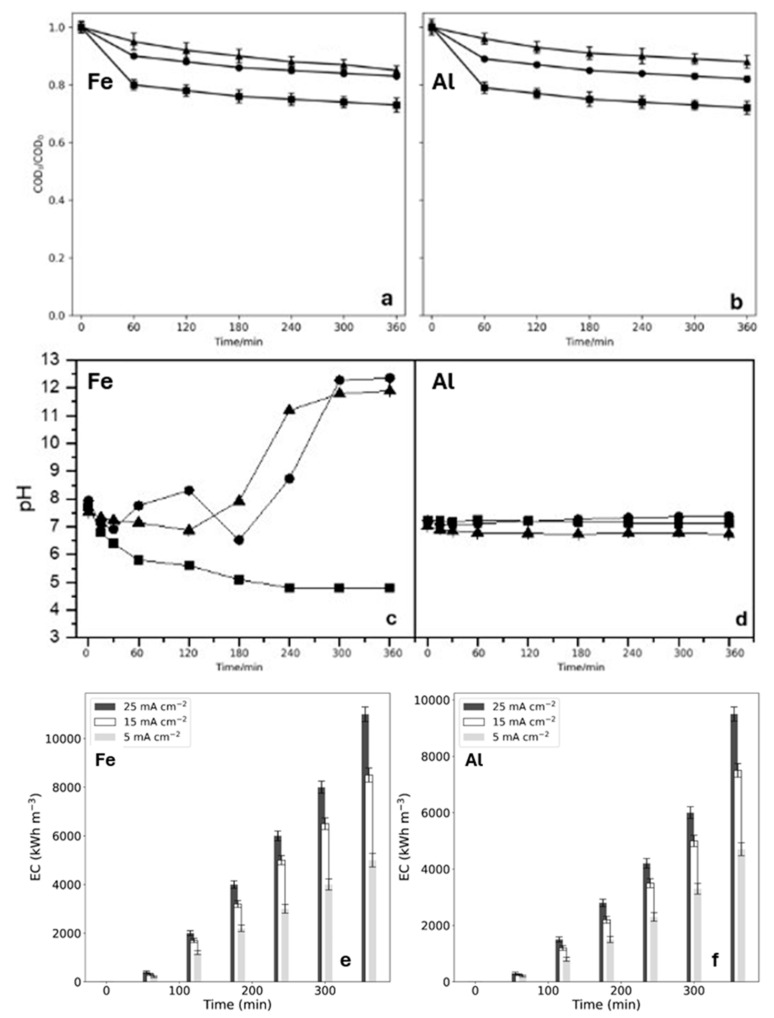
Effect of current density applied to EC on COD (**a**,**b**), pH (**c**,**d**), and $EC (**e**,**f**) with Fe and Al anodes. Experimental conditions=Methylene blue 50 mg L^1^, NaCl 50 mM (Solution B), room temperature. ■ = 25 mA cm^−2^, ● =15 mA cm^−2^, ▲ = 5 mA cm^−2^.

Current density is one of the key parameters in EC, since according to Faraday’s law, the dissolution of the anode increases as the applied current density increases. This implies a greater number of ions and therefore greater flocs that trap the dye, which favors the effectiveness of the process. For solution B (in NaCl medium) in a range between 5–25 mA cm^−2^, the effect of current density on the removal of the chemical oxygen demand (COD), pH variation, and energy consumption ($EC) was studied, since it is the electrolyte that presented the best discoloration of the solution. Figure 2a,b show the influence of current density on the COD removal with both electrodes. The results show an increase in COD removal as the current density rises. An increment in the current density implies that it favors both the production of iron and aluminum hydroxides, as well as the homogeneity of the above and of the dye molecules, promoting the separation of the flocs by flotation. However, after 60 min of treatment, the COD value becomes practically steady. This residual COD may be due to the formation of colorless persistent organic by-products or due to colored products, which do not absorb at the maximum wavelength of the dye. This would explain why the absorbance drops off completely at that wavelength, but COD removal is low [46]. After 360 min of treatment, the percentages achieved with the three current densities are less than 40%, both with Fe and Al, which is disadvantageous if the aim is to obtain a complete decontamination of the solution.

On the other hand, variation of pH over time was also studied (see Figure 2c,d) for solution B with both electrodes, and applying the three current densities. With the Fe electrode, a significant variation in pH was observed over time. At current densities of 5 and 15 mA cm^−2^, an increase in pH was observed as the electrolysis time progressed, reaching highly alkaline values, which could be due to the formation of OH^−^ ions according to Equation (7).

(7) $$2H_{2}O_{\left(l\right)}+2e^{-}\to 2H_{2(g)}+2OH^{-}$$

When the highest current density was applied, a decrease in this parameter was observed. According to these pH values, Fe(OH)_3_ predominates in high concentrations at a pH above 1.0, while Fe(OH)_2_ is found in high concentrations at a pH lower than 5.5. The Fe(OH)_3_ and Fe(OH)_2_ species currently predominate; these species decrease with the increasing alkalinity of the medium, because they do not generate hydroxyl ions. With the Al electrode, the pH remained relatively constant between values of 6.7–7.4 independent of the applied current density. These values, close to neutrality, support the possibility of reusing these waters in, for example, ornamental irrigation, since they are within the range established by Chilean legislation [47].

Finally, in relation to the $EC of the process per m^3^ of treated water (Figure 2e,f), it was determined that the higher the applied current density, the greater the energy consumption of the process, both for the Fe and Al electrodes. The cost is slightly higher during the first 120 min of the process with the Fe anode, and higher with the Al anode at the end of the EC.

Based on the results, NaCl was identified as the optimal supporting electrolyte, as it allows for complete discoloration of the solution. Since COD removal does not exceed 40% and remains practically constant after 60 min, regardless of the electrode or current density applied, an intermediate current density of 15 mA cm^−2^ was determined to be the most suitable. Regarding pH stability and energy consumption, further studies will focus on the Al electrode. After 1 h of treatment, when complete discoloration has already been achieved, energy consumption is lower for Al when compared to Fe. Additionally, the Al electrodes help maintain a relatively stable, neutral pH, offering better control over the electrochemical process.

### 2.2. Treatment of OII and SDS Solutions by EO

**Figure 3:**
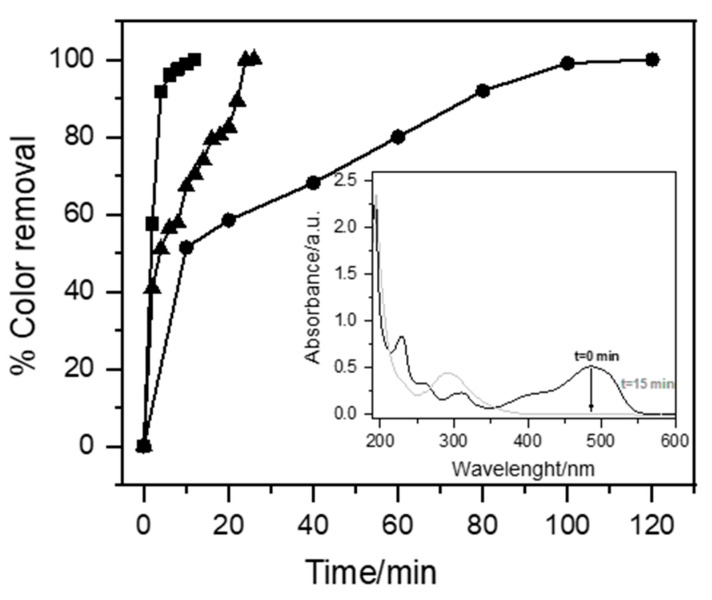
Effect of supporting electrolyte on the color removal of OII with MMO electrode by EO. Experimental conditions = Orange II 50 mg L^−1^, room temperature, 15 mA cm^−2^. ■ = NaCl 50 mM (solution D), ● = Na_2_SO_4_ 25 mM (solution C), ▲ = NaCl 2.5 mM + Na_2_SO_4_ 25 mM (solution E).

In the EO process, the chemical nature of the supporting electrolyte determines the oxidants that will be produced during the process. In this sense, the effect of NaCl, Na_2_SO_4_, and NaCl + Na_2_SO_4_ as supporting electrolytes in the removal of Orange II (OII) using MMO anodes was studied. The results show that the color of the solution was completely removed in less than 15 min when NaCl and NaCl + Na_2_SO_4_ were used as the electrolyte (inset Figure 3). For Na_2_SO_4_, the same effect was achieved almost 120 min later. The faster discoloration reached with NaCl can be attributed to the oxidation of the dye, which occurs on the surface of the electrode (Equation (1)) and within the solution because the chloride ions can be oxidized at the anode. This leads to the formation of molecular chlorine, which, because of its hydrolysis, gives rise to the formation of hypochlorous acid [48,49]. The formation of the hypochlorite ion is conditioned by pH, since at a pKa close to 8, hypochlorous acid is in equilibrium with the hypochlorite ion.

In this study, the main oxidant responsible for the oxidation of the dye in NaCl would be ClO^−^ instead of hypochlorous acid, since the pH of the solution was maintained between 8.23 and 8.64 during the process.

On the other hand, with sodium sulfate, the oxidation would not be mediated by highly oxidizing species such as the hydroxyl radical (Equation (1)), since this electrolyte resulted in the lowest decolorization; therefore, the reaction occurs only by direct oxidation on the surface of the electrode, and this is consistent with what has been reported in the literature when using MMO-type electrodes in the presence of Na_2_SO_4_ [50,51]. So, the color removal is Na_2_SO_4_ < NaCl + Na_2_SO_4_ < NaCl.

**Figure 4:**
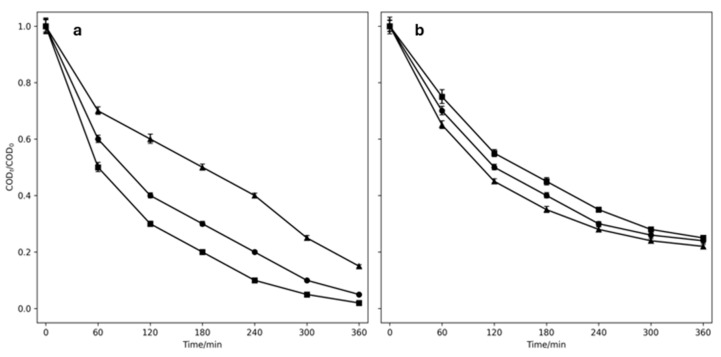
Effect of current density on the COD decay with MMO electrode using EO. (**a**) NaCl 50 mM (solution D) and (**b**) NaCl 2.5 mM + Na_2_SO_4_ 25 mM (solution E). Experimental conditions = Orange II 50 mg L-1, room temperature. ■ = 25 mA cm^−2^, ● = 15 mA cm-2, ▲ = 5 mA cm^−2^.

When NaCl (solution D) is used, it is important to also study how its presence affects the removal of COD, since the formation of chlorinated intermediates and/or by-products could interfere with this. Based on the OII color removal times, the electrolytes NaCl (solution D) and a mixture of NaCl + Na_2_SO_4_ (solution E) were compared. In NaCl (see Figure 4a) the COD decay is directly related to the applied current density, while in the electrolyte mixture it is not (Figure 4b). After 360 min of treatment, a 100% COD decay was reached with the two highest current densities in NaCl (solution D), while with these same currents, in the electrolyte mixture, a 94% COD decay (solution E) was reached at the end of the process. This confirms that NaCl not only achieves complete decolorization, but also complete mineralization; that is, it completely reduces the organic load of both the dye and the possible intermediates, and/or the reaction products formed. The lower reduction in COD in the mixture is justified by its lower concentration of chlorides, since the mineralization occurs because of the active chlorine species within the solution. Therefore, there is oxidation of the dye, but it cannot be completely transformed into CO_2_. Since there are no significant differences between the current densities of 15 and 25 mA cm^−2^ for COD reduction, studies will continue to apply the first density. These results are promising, as complete decolorization of the solution was achieved in a short electrolysis time while using only half the current density applied in previous studies [52,53].

**Figure 5:**
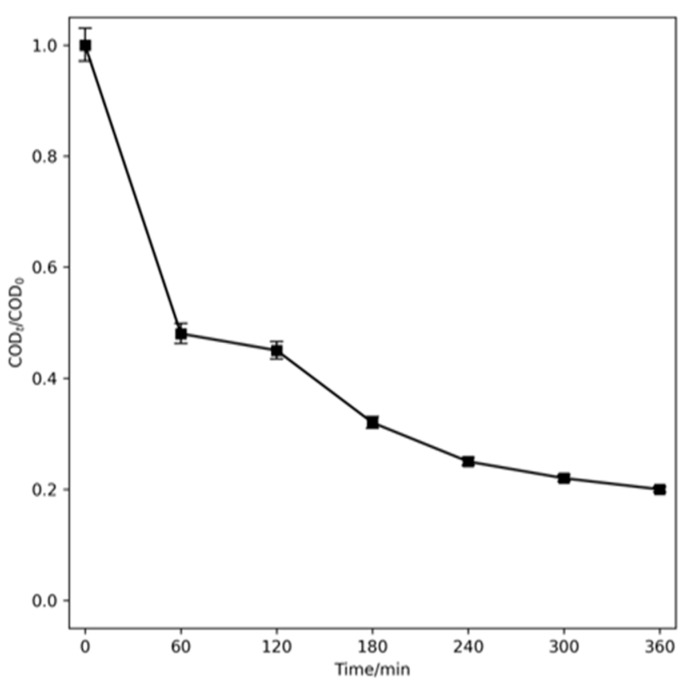
COD decay of SDS with MMO electrode using EO in NaCl 50 mM (solution F). Experimental conditions = SDS 1500 mg L^−1^, 15 mA cm^−2^, room temperature.

Based on the results obtained from the treatment of OII using EO, the SDS elimination studies using EO were carried out by applying the optimal conditions with a current density of 15 mA cm^−2^ and a NaCl electrolyte (see Figure 5) (solution F). The mineralization of this surfactant using EO is promising, since it reaches 80% after 360 min of electrolysis, considering the complexity of its structure.

**Figure 6:**
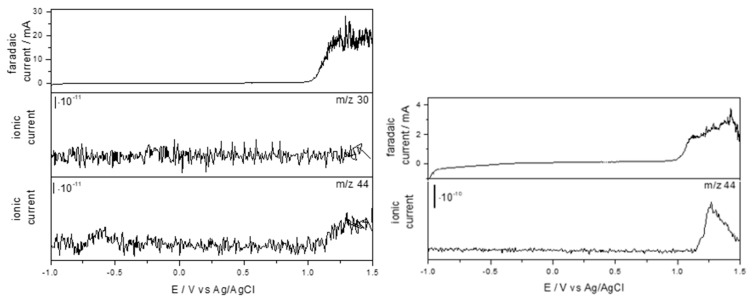
DEMS results corresponding to the MMO electrode: (**left**) OII in NaCl 50 mM (solution D) and (**right**) SDS in NaCl 50 mM (solution F). Scan rate: 1 mV s^−1^. Ionic currents calculated from DEMS as a function of applied potential for NO = (*m*/*z* = 30) and CO_2_ (*m*/*z* = 44) species.

According to the results from the DEMS analysis (Figure 6), in the presence of OII (solution D), a product with a ratio *m*/*z* = 44 is generated, from a potential of 1.14 V, while in the presence of SDS (solution F), the same product is produced from a potential of 1.156 V. The DEMS results confirmed those obtained in relation to the COD decay. For both OII and SDS (see Figure 4a and Figure 5), the formation of carbon dioxide demonstrates the mineralization of organic compounds using EO. Likewise, no product with a *m*/*z* ratio of 30 ratio is generated with OII, despite containing N atoms in its structure. It is interesting to explain that according to the National Institute of Standards and Technology, a *m*/*z* ratio of 44 may correspond to CO_2_ or N_2_O. But if CO_2_ is the product, only this mass is representative. On the other hand, if the product is N_2_O, a ratio of greater representativeness should appear at a *m*/*z* of 30, a higher intensity than the *m*/*z* ratio of 44. Therefore, the formation of NO has declined. Then, according to DEMS experiments, the gaseous product detected is CO_2_.

### 2.3. Hybrid Process to Treat Greywater

**Figure 7:**
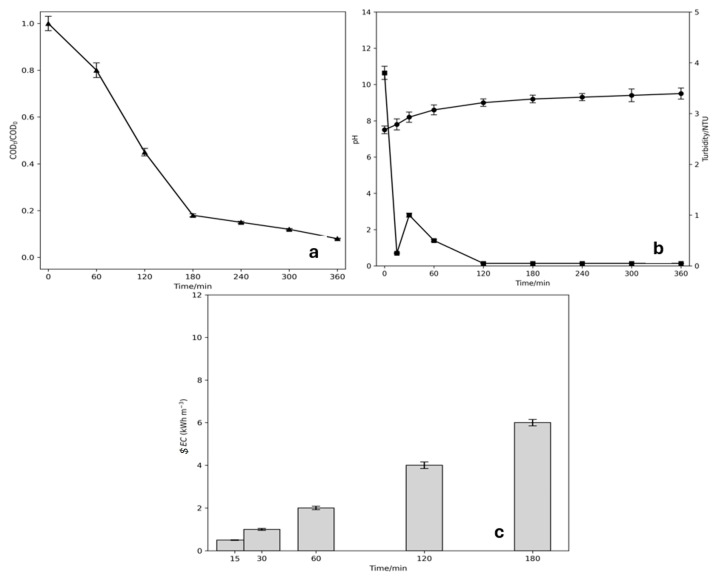
Simulated greywater treatment (solution G) using EC-EO. (**a**) COD decay, (**b**) pH and turbidity, (**c**) Energy consumption ($EC). Experimental conditions = NaCl 50 mM, washing machine water with SDS 1500 mg L^−1^ + OII 50 mg L^−1^ + MB 50 mg L^−1^, current density of 15 mA cm^−2^ and room temperature.

A sample of 1L of greywater enriched with MB, SDS and OII was treated (solution G). The same electrochemical system used in EC and EO was used with the optimal experimental conditions found (15 mA cm**^−^**^2^ and NaCl as a support electrolyte). Based on the EO, larger COD removal was achieved for both the dye and the surfactant. Short decolorization times were achieved using two MMO electrodes and one Al electrode as anodes. For this setup, three stainless steel electrodes were used as cathodes. After 180 min of hybrid treatment, a 90% COD removal (Figure 7a) and 90% color reduction were achieved, which is satisfactory considering the complexity of the matrix, and better than either of the two processes used independently. The applied current density and the nature of the anode materials are comparable with other results reported in the literature [54]. pH monitoring during electrolysis shows a variation between 7 and 10, and at the end of electrolysis, a pH value close to 8 was reached, which is within the range allowed by Chilean legislation. Furthermore, because of the mixed process, the turbidity of the sample was completely reduced after 120 min of treatment (Figure 7b). Finally, as the electrolysis time increased, the energy consumption per cubic meter of treated water (Figure 7c) rose.

### 2.4. Hybrid Process to Treat Real Washing Machine Water

Given these results, the combined process was applied to treat real washing machine water for 180 min. After this time, COD and biochemical oxygen demand (BOD_5_) decreased noticeably and reached constant levels. BOD_5_ in greywater is always lower than the COD, and the BOD_5_/COD ratio is used to assess its biodegradability. These ratios range from 0.31 to 0.71, indicating that about half of the organic matter is biodegradable [52], although values as high as 4:1 have been reported [54]. The predominance of COD is attributed to the presence of xenobiotic organic compounds from household and pharmaceutical chemicals, which are difficult to degrade and may pose environmental risks. The concentration of coliforms decreases to fecal coliform (FC) concentrations < 2 MPN/100 mL, after 15 min of treatment. Detailed data for the different electrolysis times are presented in Figures S1 and S2. The presence of fecal coliforms is practically non-existent, and greywater disinfection can be achieved in 15 min of electrolysis. This is consistent with studies by several authors, who emphasize that electrochemical technologies are designed to remove *E. coli*, coliforms, and helminth eggs [29]. This is crucial because bacteria require more than 10^3^ cells to cause an infection, creating a potential health hazard for the reuse of treated water, even after disinfection. The values obtained for the concentrations of COD, BOD_5_, anions, cations, surfactants, phenols, and fecal coliforms after treating real washing machine water are summarized in Table 1.

Nitrogen in the form of ammonia, ammonium, total nitrogen, and nitrate is an essential nutrient for plants and animals; however, an excess of this can be harmful, especially in waters, since dissolved oxygen is reduced. This depletion adversely affects aquatic life and can lead to the excessive growth of photosynthetic organisms, ultimately resulting in eutrophication. Ammonia and ammonium reached values of 0.40 ± 0.02 and 0.51 ± 0.09 mg L^−1^ before treatment and 11.6 ± 0.05 and 12.7 ± 0.03 mg L^−1^ after treatment, respectively. The concentrations of total nitrogen and nitrate found were 5.30 ± 0.04 and 23.5 ± 0.05 mg L^−1^ in the influent and 5.91 ± 0.08 and 26.1 ± 0.01 mg L^−1^ in the effluent. According to the literature, during the application of the EC process, the concentration of nitrate ions typically decreases [52,53]. However, the MMO electrodes, which represent the EO process in this system, may exert an inhibitory effect on this electrochemical reduction. In a NaCl medium, chlorine species such as hypochlorous acid are produced. This acid can react with the nitrite ions formed, generating nitrate ions. Sulfate ions were also studied, establishing a concentration of 34.3 ± 0.15 mg L^−1^ at the end of the process, which is within the limits established by both the World Health Organization and the U.S. Environmental Protection Agency. Its decrease is likely due to the oxidation reactions of the organic compounds in the aqueous matrix and the generation of sulfate radicals [53].

Chloride was the ionic compound that presented the highest concentrations, with average values of 30.2 and 70.3 mg L^−1^. The concentration of chlorine oxidizing species after 180 min of treatment was 138 ± 1.09 mg L^−1^. Some of these levels of the anion do not comply with international regulations for the reuse of treated water for toilet flushing and irrigation (>0.02–0.40 mg L^−1^ Cl^−^). In the case of on-site treatment and reuse of the water, this treated greywater could not be used. However, if the water is expected to be stored or transported for more than 24 h, additional chlorination to inactivate pathogenic microorganisms that could proliferate during this time, either by further electrochemical disinfection or direct addition of chlorine, would not be necessary. No chloramines or chlorates were found and/or detected after treatment. The removal efficiencies of anionic and cationic surfactants averaged 98.36% and 98.10%, respectively. In addition, oxalate and acetate anions are observed, but not citrate anions. These anions are characteristic of water mineralization treatments where the degradation of organic compounds and their by-products is very advanced. Their concentrations in the effluent were 3.04 ± 0.08 and 8.26 ± 0.19 mg L^−1^, respectively. Anionic surfactant concentrations above 30 mg L^−1^ and cationic surfactant concentrations above 10 mg L^−1^ can have a toxic effect on microbial communities, fish, and humans [45]. Finally, no presence of phenols was found, which would indicate that the combined process also manages to mineralize these toxic organic by-products. The indirect 4-AAP method used in this study is highly sensitive, having a detection limit of 0.001 mg L^−1^, and the Folin–Ciocalteu method, used as a comparative test, has a detection limit of 0.01 mg L^−1^ [55]. The latter has also been used for water and wastewater samples, obtaining very similar results for the total phenolic content. The synergistic effect increases the speed of the treatment by promoting the removal of the organic load (especially detergents and dyes), thus achieving a successful process in terms of the efficiency of the combined treatment.

## 3. Materials and Methods

### 3.1. Materials

Orange II (OII, dye content ≥ 85 %), methylene blue (MB, dye content ≥ 82%), sodium dodecyl sulfate (SDS, purity ≥ 97%) and 4-aminoantipyrine (purity ≥ 99%) were supplied by Sigma-Aldrich, Darmstadt, Alemania. Solutions of sodium sulfate and chloride (Na_2_SO_4_ and NaCl, analytical grade from Merck) were used as supporting electrolytes. HCl (37.00%) and H_2_SO_4_ (95.00–97.00%) were provided by Merck, and acetone (99.60%) by Soviquim, Santiago, Chile. Ultrapure water with a conductivity of 18 mΩ cm^−1^ was used for preparing solutions, except when other water quality is specified. Fe plates (99.90% purity), Al plates (99.95% purity) and stainless steel AISI304L plates were purchased from Innovametal S.A., Santiago, Chile. MMO electrodes were provided by NeoCoat, La Chaux-de-Fonds, Switzerland (Ru/Ir: 50/50%).

### 3.2. Solutions

Solutions of MB, OII, and SDS were prepared in tap water as a model of greywater from a washing machine, which uses this type and quality of water. When Na_2_SO_4_ 25 mM or NaCl 50 mM were added as supporting electrolyte to Methylene blue 50 mg L^−1^, they were named as Solution A and Solution B, respectively. For Orange II dye, when Na_2_SO_4_ 25 mM, NaCl 50 mM, or a mixture of them (NaCl 2.5 mM + Na_2_SO_4_ 25 mM) were added to an initial dye concentration of 50 mg L^−1^, they were named as Solution C, D and E respectively. Likewise, for SDS (1500 mg L^−1^) with NaCl 50 mM, the name of this mixture was Solution F. Finally, dark greywater consists of real washing machine water, which was used as received, without filtration or initial pH adjustment. It was obtained from a washing cycle that lasts 15 min after washing 7.5 kg of real dirty clothes (cotton, polyester, spandex) from a family composed of two children (ages 8 and 15) and two adults, with approximately 45 L of water and 0.15 L of Bio Frescura®, a common liquid detergent brand from Cleaner Chile S.A., Santiago, Chile.

Later, a 5 L wastewater sample from the washing machine was enriched with MB 50 mg L^−1^, OII 50 mg L^−1^, SDS 1500 mg L^−1^, and NaCl 50 mM, and this mixture was named Solution G. This selection was based on the reported concentrations of dyes and surfactants in some domestic wastewater, as well as previous studies on electrochemical treatment processes [9]. The contents of each solution used in this study are summarized in Table 2.

### 3.3. Equipment

The electrochemical experiments were performed at constant current using an Eletech power supply model HY3005E-2, Tesladelta, Santiago, Chile, which also displayed cell voltage. Differential Electrochemical Mass Spectrometry (DEMS) experiments were carried out using a Hiden HPR-40 DEMS, Warrington, United Kingdom. pH, conductivity, and total dissolved solids (TDS) were measured with a Milwaukee multiparameter probe model MW804, Szeged, Hungary. The absorption spectra were measured in an Agilent 8454 Diode-Array spectrophotometer, Massachusetts, EE. UU. For digestion, a HANNA digester model HI839800, Eibar, Spain, was used. To determine the chemical oxygen demand (COD) and biochemical oxygen demand (BOD_5_), a HANNA photometer model HI83399-02, Eibar, Spain, was used. The detection of inorganic ions, including ammonia, ammonium, total nitrogen, nitrate, free chlorine, and total chlorine, was accomplished through the utilization of reagents supplied by Hanna Instruments. The measurement of these samples was conducted using a Hanna Instruments HI83399 Multiparameter Photometer, Eibar, Spain. The turbidity of the solutions during the process was measured with a portable HANNA turbidity meter model HI98703-02, Eibar, Spain. Phenol determination was carried out by spectrophotometry using the 4-aminoantipyrine method as described in the Folin–Ciocalteu method [55]. Determination of the anionic and cationic surfactants was performed photometrically, using the HANNA photometer HI83399-02. A Thermo Scientific Dionex Aquion IC system was used to analyze chloride, phosphate, oxalate, acetate, and citrate anions in the samples, according with the standard method.

### 3.4. Electrochemical Experiments

**Figure 8:**
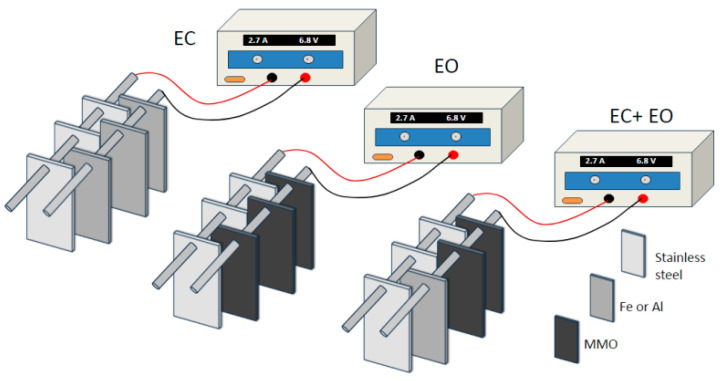
Electrodes arrangement in the EC, EO and EC+EO electrochemical systems.

Galvanostatic EC experiments were carried out in a static cell with 1.0 L of each solution under study. As mentioned previously, each salt added to the solutions, NaCl (50 mM), Na_2_SO_4_ (25 mM), or a mixture of them (NaCl 2.5 mM + Na_2_SO_4_ 25 mM), was used as a supporting electrolyte at room temperature. During the EC experiments, the solutions were stirred vigorously with a magnetic bar at 500 rpm. The sacrifice anodes were iron and aluminum plates. Three plates of Al or Fe were used as an anode and three plates of stainless steel were used as a cathode, connected in a monopolar parallel configuration (see Figure 8 for the EC electrode configuration). The geometric area of each plate was 60 cm^2^ and the interelectrode gap was 1 cm. Before each EC experiment, the Al electrodes were rinsed with acetone/ultrapure water and then immersed in 0.5 L of 1.3 M HCl solution for 5 min. Finally, the plates were washed with ultrapure water to remove surface grease and impurities present on the electrode surface. In the case of Fe electrodes, they were washed with acetone/ultrapure water mixture and immersed in a 0.1 M NaOH solution for 5 min, and finally washed with ultrapure water. After the washing process, all the electrodes were dried and weighed. At the end of electrolysis, the electrodes were rinsed thoroughly with ultrapure water to remove all solid residues from the surface, and then they were dried and weighed again. For EO, the same system was used, but three mixed metal oxide (MMO) electrodes (120 × 40 × 2 mm) were used as an anode and three stainless steel plates as a cathode. During the experimental trials, the solution was subjected to vigorous stirring with a magnetic bar at a rate of 500 revolutions per minute (rpm). In the EC + EO system, the geometric area and interelectrode gap of EC and EO were utilized in the treatment of simulated greywater. Two MMO electrodes and one Al plate were used as anodes and three plates of stainless steel as a cathode (see Figure 8).

The DEMS measurements were carried out in a type A cell coupled to the DEMS equipment, using the linear voltammetry technique in a potential window between −1.0 and 1.5 V, at a linear scan rate of 1 mV s^−1^ (2500 s per segment). MMO Ir/Ru (working electrode) (φ 5 mm rod and 5 cm length), Pt (counter electrode), Ag/AgCl (reference electrode) were used. The original solutions were diluted 10 times with ultrapure water for each measurement. In DEMS analysis, the detected signals correspond to ions separated based on their *m*/*z* values, where *m*/*z* refers to the mass-to-charge ratio of each ion. This parameter allows for the identification of gaseous products formed during electrochemical processes (e.g., H_2_ at *m*/*z* = 2, O_2_ at *m*/*z* = 32).

### 3.5. Analytical Procedures

A 1.0 L sample of Solutions A, B, C, D, E, F, and G were treated by the three electrochemical processes. For EC and EO, the effect of the applied current density (at 5, 15, and 25 mA cm^−2^) and the presence of NaCl and/or Na_2_SO_4_ as a supporting electrolyte were evaluated for the treated solutions A, B, C, D and E. For EC the nature of the anode was also studied. Solution G was treated by the EC + EO system using NaCl as a supporting electrolyte, and by applying a unique current density of 15 mA cm^−2^. The pH, conductivity, and total dissolved solids (TDS) were measured for each sample. During electrolysis, samples were withdrawn from the solution of the cell at regular time intervals, filtered, and then refrigerated at 4.0 ± 0.1 °C until the analytical procedures were performed. The decolorization (indicated as color removal in results) of the solutions were measured by their decrease of the absorbance at 664, 485 and 533 nm for MB, OII and greywater, respectively. Tap water was used as a solvent and was blank in all measurements. The measurement range depended on the sample studied in aerated solutions at room temperature. The ranges for each sample and technique are detailed next: MB, 190–800 nm; OII, 190–600 nm; SDS, 190–450 nm; and for greywater 190–450 nm. For MB, a 10x dilution was carried out to avoid saturation of the absorption spectrum. In the case of OII, a 5x dilution was required, and the SDS sample was measured as prepared without any dilution. For EO, only the OII samples were diluted by a factor of 5x.

The organic matter present in the samples was determined in the form of chemical oxygen demand (COD, colorimetric method, standard method 5220-D) and BOD_5_ (azide-modified Winkler method, NCh 2313/5 Of. 2005). Inorganic species such as ammonia, ammonium, total nitrogen, free chloride, and total chloride were detected using specific reagents with a multiparameter photometer, and other organic and inorganic anions such as chloride, nitrate, sulphate, phosphate, oxalate, citrate, and acetate were detected using an ionic chromatography (IC) system. Inorganic and organic anions present in real greywater pre- and post-electrochemical treatment were detected using IC. Greywater and anionic standard solutions were filtered and injected for identification. The separation was performed on a 4 × 0.25 mm internal diameter column Dionex IonPac AS22, Massachusetts, EE. UU. The column oven was at 30.0 °C. Manual injection was used with carbonate/bicarbonate as the mobile phase and carbonate suppressor type AERS 2 mm. The anions were identified and quantified using external standard calibration. The oxidants, including free chlorine species (Cl_2_/HClO/ClO^−^), and total chlorine were detected using the N, N-diethyl-p-phenylenediamine (DPD) colorimetric method in a photometer Hanna Instruments HI83399-02, Eibar, Spain.

In addition, the concentrations of two types of surfactants, anionic and cationic, were measured in the greywater samples. The analysis of surfactants, such as methylene blue active substances, was performed by means of the formation of a chloroform-extractable ion pair that was subsequently measured photometrically. The cationic surfactants were measured photometrically through the addition of bromophenol blue anionic dye, using a cuvette test. For the phenols analysis, the methodology is based on the oxidative coupling of phenolic compounds with 4-aminoantipyrine (4-AAP) in the presence of an oxidant, leading to the formation of antipyrine dyes. In this technique, 4-AAP reacts with unsubstituted, meta-substituted, or ortho-substituted phenolic compounds containing various functional groups such as halogen or sulphonate, methoxy, or carboxy groups. The reaction takes place at a pH of 10.0 and produces persistent red-colored antipyrine dyes. These antipyrine dyes allow the subsequent spectrophotometric determination of phenolic compounds [55].

Greywater samples were measured for biological contaminants such as fecal coliforms, which were analyzed in accordance with the protocol described in NCh 2313/22 Of. 95. Fecal coliforms were determined by means of the multiple tube technique, using the most probable number (MPN) technique.

For a better analysis of the viability of electrochemical treatments, energy consumption per unit volume and per anode mass were calculated from the equations:

(8) $$Energy\;consumption\left(\frac{kWh}{m^{3}}\right)=\frac{E\times I\times t}{V_{s}}$$

(9) $$Energy\;consumption\left(\frac{kWh}{metal\;kg}\right)=\frac{E\times I\times t}{∆m_{anode}}$$

where E corresponds to the potential (V), I the current (A), t the time (h), Vs the volume of the treated solution (L) and, Δ*m* the difference of the mass of the electrode pre and post electrolysis.

## 4. Conclusions

In conclusion, after 120 min of electrolysis at a current density of 15 mA cm^−2^, a mineralization of about 90% is obtained for real washing machine greywater, corresponding to the residual COD (126 ± 0.25 mg L^−1^), mainly composed of organic anions, chloride and sulfate, with an average removal of 98% for both anionic and cationic surfactants from real greywater, also disinfected. This suggests that the batch treatment of greywater using the two proposed techniques provides a successful response in terms of organic load removal with a residual BOD_5_ of 5.09 ± 0.31 mg L^−1^ after 180 min of treatment, and electrochemical disinfection with FC removal efficiencies equal to 99.95% after 15 min treatment time. Together they can provide a cost-effective and environmentally friendly alternative for greywater treatment and reuse. Further studies and optimization of the system to reduce energy consumption can focus on the applied current density, electrode material, and the supporting electrolyte. Future research should also emphasize testing the system in combination with other electro-processes, filtration processes, etc., that can be coupled for eventual scale-up, identifying and measuring all organic by-products generated during the process, and investigating the effects on ornamental irrigation as well, among other potential reuses in accordance with legislation.

## Figures and Tables

**Table 1:** COD, BOD_5_, ions, surfactants, total phenolic total (TPC) concentrations (mg L^−1^), and fecal coliforms (NMP/100 mL) in real washing machine water before (influent to time zero) and after (effluent to time 180 min) electrolysis using the combined process.

Parameters	Average to Time 0 min (mg L^−1^)	Average to Time 180 min (mg L^−1^)	Concentrations (Before-After Electrolysis) (mg L^−1^)
COD	1465 ± 0.02	126 ± 0.25	1466–126
BOD_5_	688.5 ± 0.15	5.09 ± 0.31	695.0–5.05
NH_3_	0.40 ± 0.01	11.6 ± 0.22	0.38–11.9
NH_4_^+^	0.51 ± 0.03	12.7 ± 0.15	0.48–12.9
TN	5.30 ± 0.07	5.91 ± 0.04	5.20–5.97
NO_3_^−^-N	23.5 ± 0.05	26.1 ± 0.19	23.1–26.3
SO_4_^2−^	510.3 ± 1.49	34.3 ± 0.34	508–34.7
Cl^−^	30.2 ± 0.25	70.3 ± 0.36	29.8–71.8
Free chlorine (as Cl_2_)	0.015 ± 0.03	138 ± 1.09	0.015–139
Total chlorine (as Cl_2_)	0.015 ± 0.03	147 ± 1.43	0.015–149
PO_4_^3−^	0.60 ± 0.02	Not found	0.58–not found (<LD)
C_2_O_4_^2−^	Not found	3.04 ± 0.01	Not found (<LD)–3.06
COO^−^	Not found	8.26 ± 0.03	Not found (<LD)–3.06
C_3_H_5_O(COO)_3_^−^	Not found	Not found	Not found (<LD)–not found (<LD)
Anionic surfactants	70.2 ± 0.18	1.15 ± 0.04	70.0–1.20
Cationic surfactants	30.5 ± 0.22	0.58 ± 0.02	30.2–0.62
TPC	Not found *	Not found *	Not found (<LD)–not found (<LD)
Fecal coliforms	4.50 × 10^3^ ± 0.02 (MPN/100 mL)	<2 (MPN/100 mL)	5.40 × 10^3^–<2 (MPN/100 mL) **

Note: TN: total nitrogen; TPC: total phenolic content; *: not found using 4-AAP and Folin–Ciocalteu methods; **: LD = 2 MPN/100 mL (NCh 2313/22 Of. 95).

**Table 2:** Solution under study.

Solution	Dye/Surfactant	Electrolyte	Water Type
A	MB 50 mg L^−1^	25 mM Na_2_SO_4_	Tap water
B	MB 50 mg L^−1^	50 mM NaCl	Tap water
C	Orange II 50 mg L^−1^	25 mM Na_2_SO_4_	Tap water
D	Orange II 50 mg L^−1^	50 mM NaCl	Tap water
E	Orange II 50 mg L^−1^	2.5 mM NaCl + 25 mM Na_2_SO_4_	Tap water
F	SDS 1500 mg L^−1^	50 mM NaCl	Tap water
G	MB 50 mg L^−1^ Orange II 50 mg L^−1^ SDS 1500 mg L^−1^	50 mM NaCl	Enriched water of real washing machine water

## Data Availability

The original contributions presented in this study are included in the article. Further inquiries can be directed to the corresponding author.

## Supplementary Materials

The following supporting information can be downloaded at: https://www.mdpi.com/article/10.3390/ijms26136445/s1.
