# Supplementary material for: Optimization of a Monopolar Electrode Configuration for Hybrid Electrochemical Treatment of Real Washing Machine Wastewater
Source: Int J Mol Sci. 2025 Jul 4;26(13):6445. doi: 10.3390/ijms26136445 (PMC12249797; doi:10.3390/ijms26136445)
Supplement: Supplementary file 1 [file ijms-26-06445-s001.zip › ijms-3735512-supplementary.pdf]

Optimization of a Monopolar Electrode Configuration for Hybrid Electrochemical Treatment of Real Washing Machine Wastewater

SUPPLEMENTARY MATERIAL

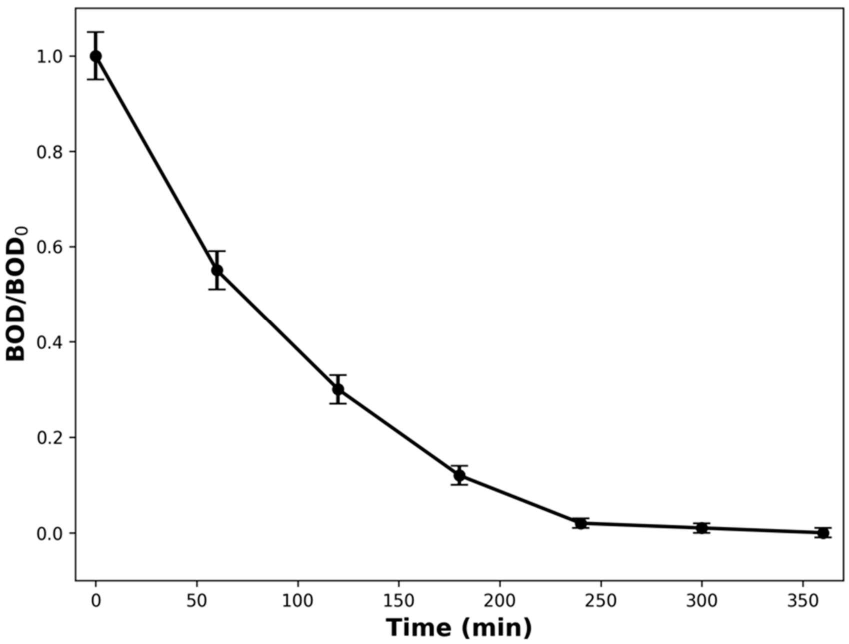

Figure S1. Real washing machine water treatment by EC–EO. BOD_5 decay. Experimental conditions: current density of 15 mA cm^{-2} and room temperature.

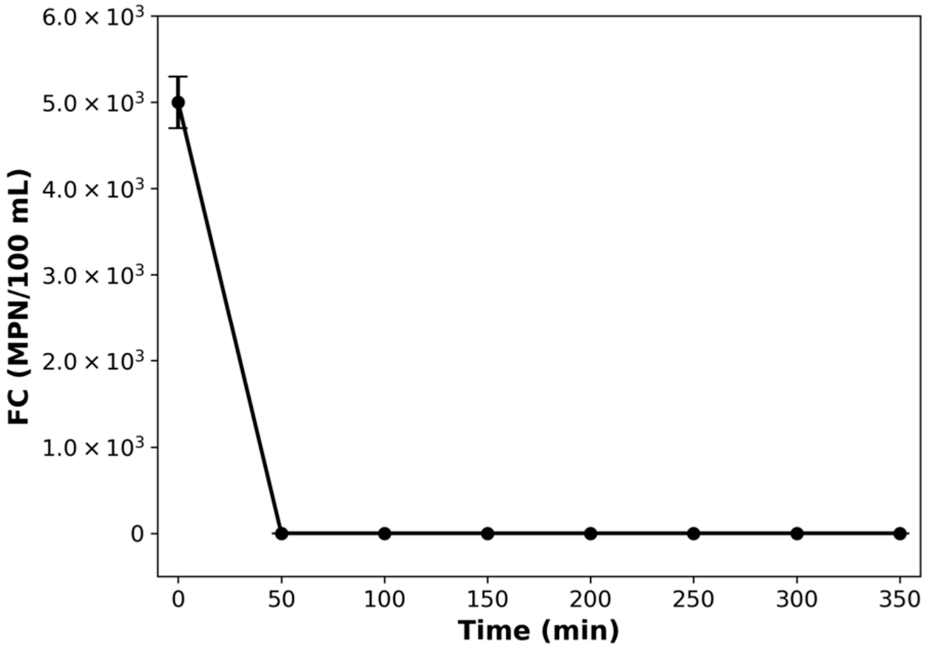

Figure S2. Real washing machine water treatment by EC–EO. Fecal coliforms (MPN/100 mL) in the treatment time. Experimental conditions: current density of 15 mA cm^{-2} and room temperature.
